# Attentionally directed oculomotor training produces disease-stage-dependent effects on plasma tau, cognition, and oculomotor signatures in patients with mild cognitive impairment

**DOI:** 10.1007/s11357-026-02474-7

**Published:** 2026-08-12

**Authors:** Ricardo Martínez-Flores, Laura Pérez Zapata, Marta Del Campo Milan, Helena Blasco-Forniés, Esther Jiménez-Moyano, Javier Torres-Torronteras, Marc Suárez-Calvet, Agustin Ibáñez, Isabel Martín-Sobrino, Neus Falgàs, Oriol Grau-Rivera, Carlos Cristi-Montero, Corinna Perone, Hans Supèr

**Affiliations:** 1 Institute of Neurosciences, University of Barcelona (UBNeuro), 08035 Barcelona, Spain; 2 Vision and Control of Action Group, Department of Cognition, Development and Educational Psychology, University of Barcelona, 08035 Barcelona, Spain; 3 Barcelonaβeta Brain Research Center (BBRC), Pasqual Maragall Foundation, 08005 Barcelona, Spain; 4 Hospital del Mar Research Institute, 08003 Barcelona, Spain; 5 Servei de Neurologia, Hospital del Mar, 08003 Barcelona, Spain; 6 Latin American Brain Health Institute (BrainLat), Universidad Adolfo Ibáñez, Santiago, Chile; 7 Global Brain Health Institute, Trinity College Dublin, Dublin, Ireland; 8 Department of Biophysics, School of Medicine, Istanbul Medipol University, 34815 Istanbul, Turkey; 9 Cognitive Neuroscience Center (CNC), Universidad de San Andrés, Buenos Aires, Argentina; 10 Alzheimer’s Disease and Other Cognitive Disorders Unit, Neurology Service, Hospital Clínic de Barcelona, Fundació de Recerca Clínic Barcelona-IDIBAPS, Universitat de Barcelona, Barcelona, Spain; 11 Centro de Investigación Biomédica en Red en Enfermedades Neurodegenerativas (CIBERNED), Madrid, Spain; 12 Hospital del Mar Research Institute, Barcelona, Spain; 13 Servei de Neurologia, Hospital del Mar, Barcelona, Spain; 14 IRyS Group, Physical Education School, Pontificia Universidad Católica de Valparaíso, Valparaíso, Chile; 15 Center for Interdisciplinary Research in Biomedicine, Biotechnology and Well-Being (CID3B), Universidad Católica de Valparaíso, Valparaíso, Chile; 16 Braingaze SL, Mataró, Spain; 17 Catalan Institution for Research and Advanced Studies (ICREA), Barcelona, Spain

**Keywords:** Mild cognitive impairment, Gamma entrainment, Oculomotor training, Phosphorylated tau, Glymphatic clearance

## Abstract

Attentionally directed oculomotor training may engage circuits implicated in gamma-band modulation and early Alzheimer’s disease (AD) pathophysiology, yet its effects across mild cognitive impairment (MCI) risk strata remain unclear. We aimed to determine whether this training, delivered through a gamified eye-tracking task, is associated with differential effects on plasma neurodegeneration biomarkers, cognitive performance, and oculomotor signatures in individuals with MCI at differential risk of conversion to Alzheimer’s dementia. Twenty-nine participants with MCI were stratified into MCI-HR (*n* = 17) and MCI-LR (*n* = 12) based on validated MoCA and MoCA-MIS cut-off scores and completed 8 weeks of daily oculomotor training. Outcomes were assessed pre/post using permutation tests, bootstrapped Hedges’ *g*, and linear mixed-effects models. Cognitive performance improved in both groups (MoCA: *g* = 1.27–2.17; MoCA-MIS: *g* = 1.02–1.68), with no between-group difference. Plasma tau diverged by stratum: MCI-LR showed reductions in pTau-181 (*g* = −0.64, 95% CI [−1.07, −0.15]) and pTau-217 (*g* = −0.72, 95% CI [−1.08, −0.36]), whereas MCI-HR showed an increase in pTau-181 (*g* = 0.82, 95% CI [0.53, 1.16]; between-group *g* = 1.31, 95% CI [0.89, 1.78], *p* = 0.001). Amyloid, GFAP, and NfL remained stable. MCI-HR showed reduced accuracy and increased reaction times (+196.4 ms, *p* < 0.001); MCI-LR maintained stable performance. Oculomotor signatures diverged between strata post-training (group × time × stimulus interactions, *p* = 0.013–0.045). In MCI-LR, oculomotor changes correlated with reductions in pTau-181 (*ρ* = −0.60, 95% CI [−0.96, −0.20]) and pTau-217 (*ρ* = −0.69, 95% CI [−0.95, −0.20]). Oculomotor training produced stratum-dependent responses: biomarker reductions and oculomotor-molecular coupling in MCI-LR, and dissociated physiological and behavioral outcomes in MCI-HR, suggesting disease-stage-specific neuroplasticity capacity linked to tau-related processes.

**Supplementary Information:**

The online version contains supplementary material available at https://doi.org/10.1007/s11357-026-02474-7.

## Introduction

Oculomotor dysfunction provides a sensitive window into early Alzheimer’s disease (AD), reflecting disruptions in attentional, executive, mnemonic, and arousal systems that precede overt dementia [1–3]. Measures such as saccadic latency, antisaccade errors, fixation instability, and pupillary dynamics capture early changes in frontoparietal, hippocampal, and brainstem circuits before cognitive symptoms become apparent [2, 3]. The locus coeruleus (LC) is particularly relevant: it is among the earliest sites of tau pathology in Braak staging [4], regulates cortical arousal, and projects through noradrenergic pathways to oculomotor structures including the superior colliculus and Edinger-Westphal nucleus, coordinating vergence and pupillary responses that serve as functional indices of attentional network integrity [5–7]. Therefore, oculomotor and pupillometric markers may improve early diagnosis, disease staging, and monitoring of intervention effects including gamma entrainment-based approaches [1–3, 8, 9], especially in mild cognitive impairment (MCI), which represents the symptomatic yet pre-dementia stage of the AD continuum and constitutes the window in which disease-modifying strategies have the greatest potential [10]. Task-evoked vergence and pupillary signatures have been shown to correlate with plasma tau biomarker burden and to discriminate biologically defined AT(N) profiles at the individual level [8, 9], supporting their potential as functional indices of the tau-related pathological processes that gamma-based interventions aim to modify.

Forty-Hz gamma-frequency sensory stimulation has emerged as a promising non-pharmacological approach to modify AD pathology. Animal research demonstrated that visual and multisensory stimulation at gamma frequency reduces amyloid and tau burden and sustains cognitive function [11, 12], and that multisensory 40 Hz stimulation promotes glymphatic clearance of amyloid and tau through vasoactive intestinal peptide interneuron activation and increased cerebrospinal fluid influx [13]. In human trials, gamma sensory stimulation has shown cognitive and neuroimaging benefits in MCI and mild AD with favorable tolerability profiles sustained over extended periods [14, 15]. These effects appear contingent on the integrity of gamma-generating circuits: parvalbumin-positive fast-spiking interneurons in deep cortical layers are selectively vulnerable in AD, and their dysfunction may compromise the brain's capacity to respond to gamma entrainment [16, 17]. Critically, the capacity to benefit from such stimulation may also depend on disease stage, given that glymphatic efficiency deteriorates with aging and AD progression [18, 19], establishing a biological rationale for examining whether the effects of gamma-based interventions differ across the MCI spectrum.

Prior studies have induced gamma activity predominantly through passive flicker stimulation or transcranial alternating current [13–15], without exploiting a potentially more direct route: the oculomotor system, which is both selectively compromised in early AD and anatomically embedded in the same LC-noradrenergic and frontoparietal circuits implicated in gamma generation [20–23]. This anatomical overlap suggests that actively engaging the oculomotor system through attentional tasks requiring coordinated eye movements and pupillary responses may constitute an endogenous pathway to modulate gamma-related circuit activity, rather than relying on passive sensory entrainment from outside. In support of this hypothesis, microsaccades promote gamma-band synchrony in early visual areas [24] through cortical phase-reset mechanisms [25], and smooth pursuit eye movements produce sustained gamma-band activation in frontal eye fields, ventral intraparietal sulcus, and occipital areas within the 40 Hz range implicated in AD-related clearance mechanisms [11, 12, 22]. This oculomotor-gamma coupling is contingent on attentional task demands; frontal eye fields exert top-down control over gamma oscillations in occipitoparietal cortex specifically during active attentional engagement [21], and the superior colliculus generates gamma oscillations in response to attentional signals rather than passive visual input [20]. Furthermore, phasic LC discharge drives stimulus-induced gamma oscillations in prefrontal cortex alongside pupillary dilation [23, 26], providing a mechanistic link between oculomotor engagement, noradrenergic arousal, and the pupillary dynamics observed during cognitively demanding paradigms such as the oddball task. Together, these converging lines of evidence support the testable hypothesis that attentionally directed oculomotor training may recruit LC-oculomotor circuits through an active, endogenous mechanism capable of engaging the same gamma-band pathways targeted by passive sensory stimulation paradigms. If this mechanism is operative, engagement of prefrontal and frontoparietal attentional circuits through LC-gamma pathways would be expected to promote glymphatic clearance of phosphorylated tau and to improve cognitive performance through sustained recruitment of the executive and mnemonic networks most impaired in the MCI prodrome. Whether this endogenous route translates into modifications in neurodegeneration biomarkers or cognitive function in MCI, and whether its efficacy is constrained by disease stage, remains to be established.

Despite this growing evidence base, three critical gaps remain unaddressed. First, existing gamma stimulation trials have administered sensory exposure passively, without integrating it into an active protocol that engages attentional control circuits; since frontoparietal and prefrontal networks are the primary sites of endogenous 40 Hz gamma generation in humans and among the most affected in MCI, paradigms that bypass these circuits may partly account for the more modest human effects relative to animal findings. Second, no study has jointly characterized intervention effects on CSF or blood-based neurodegeneration biomarkers, cognitive performance, and task-evoked oculomotor signatures as an integrated multimodal readout. Third, prior studies have not examined whether intervention effects differ as a function of cognitive risk stratum within the MCI spectrum, leaving unresolved whether biomarker and functional responses are homogeneous or reflect qualitatively distinct profiles. Therefore, this study aimed to determine whether attentionally directed oculomotor training, administered through a gamified eye-tracking task engaging the 40 Hz-relevant LC-oculomotor-frontoparietal circuit, is associated with differential effects on plasma neurodegeneration biomarkers, cognitive performance, and oculomotor signatures in individuals with MCI at differential risk of conversion to Alzheimer’s dementia.

We hypothesized that, by targeting frontoparietal attentional circuits through an endogenous LC-gamma mechanism, the intervention would promote phosphorylated tau clearance and improve cognitive performance in individuals with sufficiently preserved circuit integrity. Accordingly, the intervention was expected to be associated with reductions in plasma neurodegeneration biomarkers, improvements in MoCA and MoCA-MIS performance, and enhanced oculomotor-attentional signatures, with all three effects modulated by cognitive risk stratum. Specifically, if differential gamma-generating circuit integrity and glymphatic clearance capacity across the MCI spectrum constrain the responsiveness to attentionally directed oculomotor engagement, participants with lower conversion risk would be expected to show a more coherent and favorable response pattern than those at higher risk.

## Methods

This study employed a non-randomized, assessor-blinded, pre–post intervention design in which all participants received the same intervention and were stratified into two analytically defined subgroups based on baseline cognitive status. A parallel control group was not included for two reasons. First, the primary aim of this study was to characterize the biological and oculomotor response profile of the intervention across disease-risk strata, for which a within-subject pre–post design with maximum statistical power within the available sample was prioritized. Second, a prior pilot study using the same gamified eye-tracking paradigm in an MCI population with an active control group did not show significant cognitive or pupillary improvements in the control condition [27], providing preliminary justification for the interpretation that the observed effects are not attributable solely to non-specific engagement or repeated assessment exposure. The trial is registered at ClinicalTrials.gov (ID: NCT06963879) and was approved by the Bioethics Committee of Hospital Universitari de Bellvitge (code: PID2022-139968OB-I00). Participants were unaware of their subgroup assignment, as were all outcome assessors (psychologists, eye-tracking personnel, and blood extraction staff). Only intervention personnel were aware of each participant’s cognitive stratum, and statistical analyses were performed blind to group assignment. Reporting followed the TREND Statement recommendations for non-randomized evaluations [28], and the completed TREND checklist is provided as supplementary material. All procedures were conducted in accordance with the Declaration of Helsinki and its subsequent revisions. All participants and their family members or legal representatives were informed of the study procedures and provided written informed consent prior to enrolment.

### Participants

All participants were recruited from two residential care facilities (Sant Roc and Miramar, with the same administration) located in Canet de Mar, Barcelona, Spain. As residents of these facilities, participants shared a standardized daily routine encompassing meal schedules, structured activities, and sleep-wake cycles, providing a high degree of environmental homogeneity across the sample. Of the 30 individuals initially recruited, one was excluded prior to baseline assessment due to an intercurrent medical condition unrelated to the intervention, resulting in a final sample of 29 participants who completed the full study protocol.

Participants were stratified into two subgroups based on validated MoCA and MoCA-MIS cut-off scores associated with differential short-term risk of conversion to Alzheimer’s dementia [29]: a lower AD-conversion-risk profile (MCI-LR: MoCA ≥ 20 and MoCA-MIS ≥ 7, *n* = 12) and a higher AD-conversion-risk profile (MCI-HR: MoCA < 20 or MoCA-MIS < 7, *n* = 17). All participants had received a clinical MCI diagnosis from their treating physicians according to core clinical criteria [10]. Cognitive rather than biological stratification was adopted in alignment with the clinical basis of recruitment: participants were enrolled on clinical grounds, and plasma biomarkers were collected as outcome measures, not as screening criteria; using biomarker thresholds for stratification would therefore have introduced circularity into the primary analyses. The labels MCI-LR and MCI-HR refer exclusively to cognitively-defined AD-conversion-risk strata and do not imply biologically confirmed disease stages [29]. 

Concomitant medications with potential effects on cognition and pupillary function, including cholinesterase inhibitors, memantine, anticholinergic agents, and opioids, were recorded for all participants and are reported in Table 1.

**Table 1:** Baseline participant characteristics

Variable	All	MCI-HR	MCI-LR	*p*-value
Age (y)	77.5 ± 8.59	79.7 ± 7.22	74.4 ± 9.69	0.102
Sex M/F	13/16	4/13	9/3	**0.005**
Medication *n* (%)	20 (69%)	13 (76.5%)	7 (58.3%)	0.422
MoCA	16.2 ± 5.45	12.8 ± 4.50	21.0 ± 1.56	** < 0.001**
MoCA-MIS	6.55 ± 3.55	4.17 ± 2.62	9.91 ± 0.99	** < 0.001**
Aβ42	8.14 ± 3.07	7.41 ± 2.30	9.17 ± 3.78	0.130
Aβ40	149 ± 61.3	132.9 ± 32.5	170.8 ± 80.4	0.101
Aβ42/40 ratio	0.05 ± 0.09	0.05 ± 0.01	0.05 ± 0.08	0.984
p-tau181	31.7 ± 15.5	28.4 ± 10.1	36.3 ± 20.6	0.182
p-tau 217	0.50 ± 0.26	0.56 ± 0.30	0.41 ± 0.19	0.166
GFAP	109 ± 47.4	119.7 ± 53.8	94.3 ± 32.9	0.158
NfL	29.0 ± 17.4	27.8 ± 16.1	30.4 ± 19.6	0.700
Training sessions	39.2 ± 0.78	39.2 ± 0.83	39.2 ± 0.75	0.962
Score	846,851.7 ± 71.830	837,829.4 ± 64,408.4	859,633.3 ± 81,282.5	0.431

Blood-based biomarkers in pg/ml. In bold: significant *p*-value from chi-square test for categorical variables and *t*-test for continuous variables

*y*, years; *M/F*, male/female

### Inclusion/exclusion criteria

Participants were eligible if they (1) were aged 65 years or older; (2) had sufficient visual acuity, with or without optical correction, to discriminate stimuli presented on screen; and (3) were capable of providing written informed consent, directly or through a legally authorized representative where required by the degree of cognitive impairment.

Participants were excluded if they presented with the following: (1) severe cognitive impairment, according to their treating physicians; (2) a neurological condition with documented cognitive sequelae other than MCI or AD; (3) a current major psychiatric diagnosis; (4) structural brain lesions on neuroimaging judged to be a primary contributor to cognitive deficits, such as intracranial tumors or large vascular lesions; (5) ophthalmological pathology that would preclude reliable task performance, including blindness, strabismus, nystagmus, or clinically significant retinal or oculomotor disease; or (6) insufficient proficiency in Spanish, either oral or written, to understand and complete the protocol.

### Sample size

Sample size was estimated in G*Power for a two-group, two-timepoint repeated-measures design (*f* = 0.47 from prior work [27], *α* = 0.05, power = 0.80, correlation = 0.5, *ε* = 1), yielding a minimum of 26 participants. Accounting for ~ 15% expected dropout, enrollment was set at 30. This estimation was based on the only available prior study employing the same gamified training in MCI [27], which used a simpler between-group design rather than the linear mixed-effects models applied here. A simulation-based power analysis calibrated to the full mixed-effects structure with group-by-time interactions was not feasible at the time of design, as no prior data with the required variance components and random-effects architecture existed. Given these design constraints, all mixed-effects and stratified analyses were conducted using permutation-based inference, bias-corrected and accelerated (BCa) bootstrap for effect estimation and leave-one-subject-out analysis to evaluate effect stability, minimizing dependence on asymptotic assumptions (see “Statistical analysis” section); all such findings are interpreted as hypothesis-generating.

### Oculomotor training (game)

The intervention consisted of an interactive eye-tracking game developed by Braingaze SL, designed to run on a Tobii-5L eye tracker. The task promotes saccades, antisaccades, microsaccades, cognitive vergence, smooth pursuit, and pupillary responses through top-down attentional control demands. The game has shown promising effects on cognitive performance and pupillary responses in a prior pilot study in MCI [27].

The game comprised five levels of progressively increasing difficulty (see Supplementary Figure S1). In the first level, a static bullseye target (~ 4° visual angle) appeared at random locations; participants fixated it to earn points and withheld gaze from distractor owl images for 2 s (gaze toward distractors incurred point deduction). In subsequent levels, stimuli moved horizontally, and multiple targets and distractors were presented simultaneously at increasing speeds. Progression required reaching a minimum score threshold per level; unlocked levels remained accessible throughout.

Training sessions lasted 10 min per day, 5 days per week, over a period of 8 weeks. When a participant was unable to complete a session on a given day, the missed time was recovered in the following session to ensure that all participants received a comparable intervention dose in terms of frequency and total volume.

### Outcomes

#### Blood-based biomarkers

Venous blood was collected into 10 ml EDTA tubes (BD Hemogard K2EDTA; cat. no. 367525), inverted 5–10 times, rested 30 min, and centrifuged at 1800 × g for 10 min at room temperature. Plasma was aliquoted (0.5 ml; Sarstedt ref. 72.730.105) and stored at −80 °C.

Plasma concentrations of pTau-181, Aβ40, Aβ42, GFAP, and NfL were quantified at the BarcelonaBeta Brain Research Center using single-molecule array (Simoa) technology on a Simoa HD-X Analyzer (Quanterix). Specifically, pTau-181 was measured with the Simoa pTau-181 Advantage v2.1 commercial kit (Quanterix, ref. 104111), while Abeta40, Abeta42, GFAP, and NfL were simultaneously quantified using the Simoa Neurology 4-PLEX E Advantage kit (Quanterix, ref. 103,670). Plasma pTau-217 concentrations were determined using the Simoa ALZpath p-Tau 217 v2 commercial kit (Quanterix, ref. 104371).

#### Cognition and executive function

Cognitive function and executive performance were assessed using the MoCA, a validated and widely used screening instrument sensitive to mild cognitive deficits across multiple domains including attention, executive function, language, visuospatial abilities, and delayed recall [30]. Memory-specific performance was additionally evaluated using the MoCA-MIS, a derived index that quantifies delayed free recall performance within the standard MoCA protocol and has demonstrated utility as a sensitive marker of amnestic impairment in MCI populations [29].

Of note, all cognitive assessments were conducted by trained personnel holding official MoCA certification, in strict accordance with the standardized administration and scoring guidelines established by the MoCA certification program, ensuring procedural consistency and reliability of scores across evaluation timepoints.

#### Oculomotor signature and behavioral performance

A visual oddball paradigm was administered in a dedicated room under controlled lighting (overhead lights off, curtains drawn). Participants were seated approximately 50 cm from the stimulus display screen, with the Tobii-5L remote eye-tracker positioned below it. Use of corrective lenses was permitted, and a chinrest was employed to restrict large head movements and ensure stable gaze recording throughout the session.

The oddball paradigm was selected given its well-documented capacity to activate multiple brain regions and to engage the LC-noradrenergic system, making it particularly suited to the assessment of oculomotor indices of attentional processing [31]. The task consisted of a sequence of 120 trials. Each trial began with a 2000 ms grey mask screen, followed by a centrally presented visual stimulus displayed for 2000 ms (see Supplementary Figure S2). Stimuli were strings of 11 randomly selected alphanumeric characters (upper or lower case, avoiding meaningful words or acronyms to prevent semantic bias). Stimulus strings were identical in all physical properties except color: 80% were presented in blue font and served as distractors, while the remaining 20% appeared in red font and constituted targets. Stimuli were presented in pseudorandom order. Participants were instructed to fixate on the screen and press a response button exclusively upon detection of red character strings. Instructions were repeated verbally by the examiner when necessary during the task.

Prior to the experimental sequence, the eye-tracking system was calibrated using a standard five-point binocular calibration procedure for each participant. Eye movement data were recorded continuously throughout the task, which had a total duration of approximately 6 min.

Behavioral performance during the oddball task was additionally recorded as a secondary outcome. Accuracy was operationalized as the proportion of correct responses: for target trials, a correct response required a button press within the 2000 ms stimulus window; for distractor trials, correct performance required response omission. Reaction time was computed as the latency from stimulus onset to button press for correctly detected target trials only. Both measures were analyzed trial-level alongside oculomotor features.

#### Clinician-rated observational assessment

A brief observational questionnaire was administered independently, anonymously, and blindly to the two resident psychologists (A: *n* = 19; B: *n* = 10) at intervention end. The five items (motivation and participation, attention and concentration, mood and well-being, memory and daily orientation, and social interaction and initiative) were rated on a 1–5 scale (1 = no change; 5 = very notable change) using study codes. The instrument was non-validated and designed solely to capture the perspective of trained staff with daily participant contact.

### Procedure

Blood-based biomarkers, cognitive performance, and oculomotor signatures were assessed at two timepoints: 1 week before the start of the 8-week training period (pre-intervention) and 1 week after its completion (post-intervention). All assessment sessions were supervised by trained professionals responsible for the technical application of each measure, with an additional team member present throughout to ensure that all protocols were carried out in a standardized manner across participants and timepoints.

Blood extractions were conducted on Monday mornings (08:00–09:00 h, fasting) in participants’ own rooms to minimize circadian variability and procedural stress, MoCA/MoCA-MIS assessment on Tuesday (09:00–12:00 h), and oculomotor recording on Wednesday within the same window. Assessment sessions were thus scheduled on separate days and kept independent from the blood extraction session to avoid fatigue-related confounds. This 3-day assessment schedule was applied identically at both the pre- and post-intervention timepoints.

Training sessions were conducted individually at the facility (10:00–12:00 h) in a private room with controlled luminance, with one team member present to monitor performance, verify equipment functioning, and provide standardized feedback.

### Preprocessing

Eye-tracking data were preprocessed following a rigorous protocol that included interpolation of missing data points and exclusion of trials with more than 30% interpolated data. A MAD (median absolute deviation) filter with a threshold of 3× MAD was applied to identify and exclude trials with outlier changes following previous recommendations [32]. Among the accepted trials, signal loss was minimal: 98.2% of trials required less than 5% interpolation, and only 1.6% fell in the 20–30% range; thus, the overall mean missing sample ratio was 0.4%.

Vergence angle was computed using a vector-based geometric approach from three-dimensional eye position coordinates. Two-dimensional gaze coordinates were first converted to physical dimensions (millimeters) using the monitor’s specifications (340 mm × 190 mm). Gaze direction vectors were constructed from each eye’s spatial position to its corresponding point of regard on the screen. Vergence angle (*θ*) at each timepoint was calculated as the angle between left and right gaze vectors using vector dot product geometry, expressed in degrees; thus, relative vergence changes were computed as *υ*(*t*) = (*γ*(*t*) − *γ*₀), where *γ*₀ represents baseline vergence; this method was previously published [33]. For pupil responses, absolute diameter changes were calculated as *π*(*t*) = *p*(*t*) − *p*₀, where *p*(*t*) represents the mean of left and right pupil diameters. Both baseline values (*γ*₀ and *p*₀) were calculated as the mean of the first 8 temporal points (≈242 ms pre-stimulus period), following previous recommendations [32]. Both vergence and pupil signals were temporally smoothed using a Gaussian moving-average convolution with zero-phase prior to baseline correction to optimize the signal-to-noise ratio and to ensure comparability with previous studies [8, 9]. To quantify the functional characteristics of ocular responses as proxies of attentional networks, six features were extracted from each individual trial’s vergence and pupil response (initial, global, and late slopes; area under the curve; time to peak and peak power), as previously published [8, 9]. All preprocessing was performed in Python 3.x.

### Statistical analysis

The primary outcomes were plasma p-tau181 and p-tau217, designated a priori as the central molecular outcomes based on their established sensitivity to early AD-related tau phosphorylation dynamics [34, 35] and their direct relevance to the hypothesized biological mechanism of the intervention. Vergence global and late slopes and pupil global and late slopes during target processing were additionally designated as primary oculomotor outcomes, based on prior evidence from the same oddball paradigm demonstrating that slope-based features are the most sensitive indices of AD CSF biomarker burden and AT(N) biological profile discrimination at the participant level [8, 9]. Secondary outcomes included MoCA and MoCA-MIS. All remaining oculomotor features, behavioral accuracy, and reaction time are treated as secondary; integrative cross-domain correlation analyses are treated as exploratory throughout. Analyses of the pre-specified primary outcomes, including their group-by-time(-by-stimulus) interactions, constitute the confirmatory component of this study; all other analyses are exploratory and hypothesis-generating.

Pre- to post-intervention effects on blood-based biomarkers and cognitive measures were assessed within and between cognitive risk strata using nonparametric Freedman-Lane permutation tests (10,000 permutations), preserving the hierarchical data structure within participants. Medication use with potential effects on cognition and pupillary function (anticholinergic and cholinergic agents) was retained as a covariate in all models regardless of significance given its physiological relevance. Between-group differences in pre-to-post change were additionally tested using Freedman-Lane permutation on a linear model of individual deltas (post minus pre) regressed on group and medication, permuting residuals of the medication-only reduced model across participants. Multiple comparisons were controlled via max-t family-wise error correction using the same permutation draws. Effect sizes were estimated as Hedges’ *g* with 95% BCa bootstrap confidence intervals (10,000 iterations).

Oculomotor features and behavioral outcomes of the oddball task (accuracy and reaction time) were analyzed using linear mixed-effects models with a full factorial fixed-effects structure: group (MCI-LR vs. MCI-HR) × time (pre vs. post) × stimulus (distractor vs. target), with correlated random slopes for time by participant, following Barr et al. [36]; MCI-LR, pre-intervention, and distractor were the reference levels. Reaction time was modeled with the same group × time fixed-effects structure but restricted to correct target trials only, which reduced observations per participant per timepoint to approximately 24; a random slope for time produced singular fits across all optimizer configurations and was therefore replaced by a random intercept only. Given the small sample size, inference for two or three interaction terms constituting the primary hypotheses did not rely on Wald approximations: *p*-values were obtained via Freedman-Lane permutation tests (5000 permutations), permuting conditional residuals of a reduced model within participants to preserve the hierarchical data structure, and using the absolute t-statistic of the full model as the test statistic, as previously recommended for permutation inference in mixed-effects models [37, 38]; max-t family-wise error correction was applied consistently across all outcomes as described above. 95% CIs were estimated via BCa cluster bootstrap (5000 iterations), resampling participants as the exchangeable unit of inference with jackknife acceleration, consistent with standards for bootstrap inference under hierarchical data structures [39]. Effect stability for all significant findings was examined post-hoc via leave-one-subject-out analysis, verifying absence of sign reversals or changes exceeding two standard deviations in any reported effect; all significant effects were stable, see supplementary material (Table S1).

Age and sex were excluded as their inclusion compromised model convergence under the correlated random slopes structure, which was prioritized given its necessity for valid inference [36]. Baseline MoCA was not considered given its collinearity with the Group factor. Medication use was evaluated in a sensitivity analysis; unlike the permutation-based models for biomarkers and cognition where it was partialed out via the Freedman-Lane procedure without additional model complexity, its inclusion in the LMMs consumed degrees of freedom inconsistent with the sample size constraints motivating the resampling approach throughout. All significant interactions remained stable in direction, magnitude, and significance, and medication was non-significant in every model. Full sensitivity results are provided in the Supplementary Material (Table S2). Given the significant sex imbalance between strata (Table 1), sex was similarly evaluated as a covariate for all significant oculomotor, biomarker, and cognitive effects; all interactions remained stable in direction, magnitude, and significance, and sex was non-significant in every model (Supplementary Tables S3, S4).

To integrate oculomotor findings with biomarker and cognitive outcomes, best linear unbiased predictors (BLUPs) of participant-specific random slopes for Time were extracted from models showing significant time interactions [40], including both stimulus conditions to capture overall oculomotor change without circularity, as plasma and cognitive outcomes were external to the oculomotor models. BLUPs of the random intercept for participant were additionally extracted from the accuracy and reaction time models to provide a comprehensive individual-difference index of behavioral change for inclusion in the integrative correlation analyses. BLUPs were correlated with pre-to-post changes in plasma and cognitive outcomes using Spearman rank correlations stratified by group, where change was operationalized as the residual from regressing post-intervention ranks on pre-intervention ranks within each group, consistent with the rank-based ANCOVA framework [41]. BCa bootstrap confidence intervals (10,000 iterations) were computed for each rho. These analyses are exploratory given the small subgroup sizes.

Descriptive analyses only (median, IQR, percentage distribution) were conducted for the non-validated observational questionnaire. All analyses were performed in RStudio.

## Results

Of the 30 individuals screened, 29 were enrolled. One was excluded prior to baseline assessment due to an intercurrent medical condition and contributed no data. All 29 enrolled participants completed the intervention and post-intervention assessment and were included in the primary analyses. No protocol deviations occurred.

Table 1 presents the baseline characteristics of the full sample and by cognitive group. Both groups were comparable in age, medication consumption, plasma biomarkers levels, training adherence, and accumulated game score, with no statistically significant differences on any of these variables. The only significant between-group differences at baseline were those defining the stratification itself, global cognitive performance and memory-specific performance as indexed by the MoCA and MoCA-MIS and sex distribution, with a higher proportion of women in the MCI-HR group, although sex did not contribute significantly as a covariate in any of the statistical models and was excluded from final analyses. These characteristics confirm adequate baseline comparability across all variables not used for group allocation.

Results are presented in order of clinical relevance, beginning with plasma-based and cognitive outcomes analyzed, followed by ocular biomarkers derived from mixed-effects models, and concluding with integrative analyses.

### Effects of oculomotor training on plasma-based biomarkers

Plasma-based biomarkers of neurodegeneration were analyzed using Freedman-Lane permutation tests with medication as a covariate, both in the full sample and stratified by cognitive state (MCI-HR vs. MCI-LR).

No significant pre–post changes were observed for plasma Aβ40, Aβ42, the Aβ42/Aβ40 ratio, NfL, or GFAP, either in the full sample or when analyses were stratified by cognitive status. Likewise, no significant between-group differences in pre-to-post change were detected for these biomarkers. Detailed results for these non-significant outcomes are provided in the Supplementary Material (Table S5).

In contrast, significant and differential effects emerged for phosphorylated tau biomarkers. For p-tau181, analyses of the full sample did not reveal a uniform pre-to-post change. Stratified analyses demonstrated divergent trajectories: MCI-HR exhibited a significant increase in p-tau181 following the intervention (Hedges’ *g* = 0.82, 95% CI [0.53, 1.16], *p* < 0.001), whereas MCI-LR showed a significant reduction (Hedges’ *g* = −0.64, 95% CI [−1.07, −0.15], *p* = 0.031). The between-group comparison of pre-to-post change confirmed this divergence as large and statistically significant (Hedges’ *g* = 1.31, 95% CI [0.89, 1.78], *p* = 0.001), indicating that the difference in trajectories was not attributable to baseline differences between groups and persisted after controlling for medication.

For p-tau217, a non-significant trend toward reduced levels was observed in the full sample (Hedges’ *g* = −0.18, 95% CI [−0.54, 0.21]). Stratified analyses revealed a significant pre-to-post reduction in MCI-LR (Hedges’ *g* = −0.72, 95% CI [−1.08, −0.36], *p* = 0.010), whereas no significant change was detected in MCI-HR (Hedges’ *g* = 0.07, 95% CI [−0.54, 0.44]). The between-group difference in pre-to-post change did not reach statistical significance (Hedges’ *g* = 0.23, 95% CI [−0.46, 0.94], *p* = 0.631), consistent with the divergence being driven primarily by p-tau181 dynamics.

**Fig. 1:**
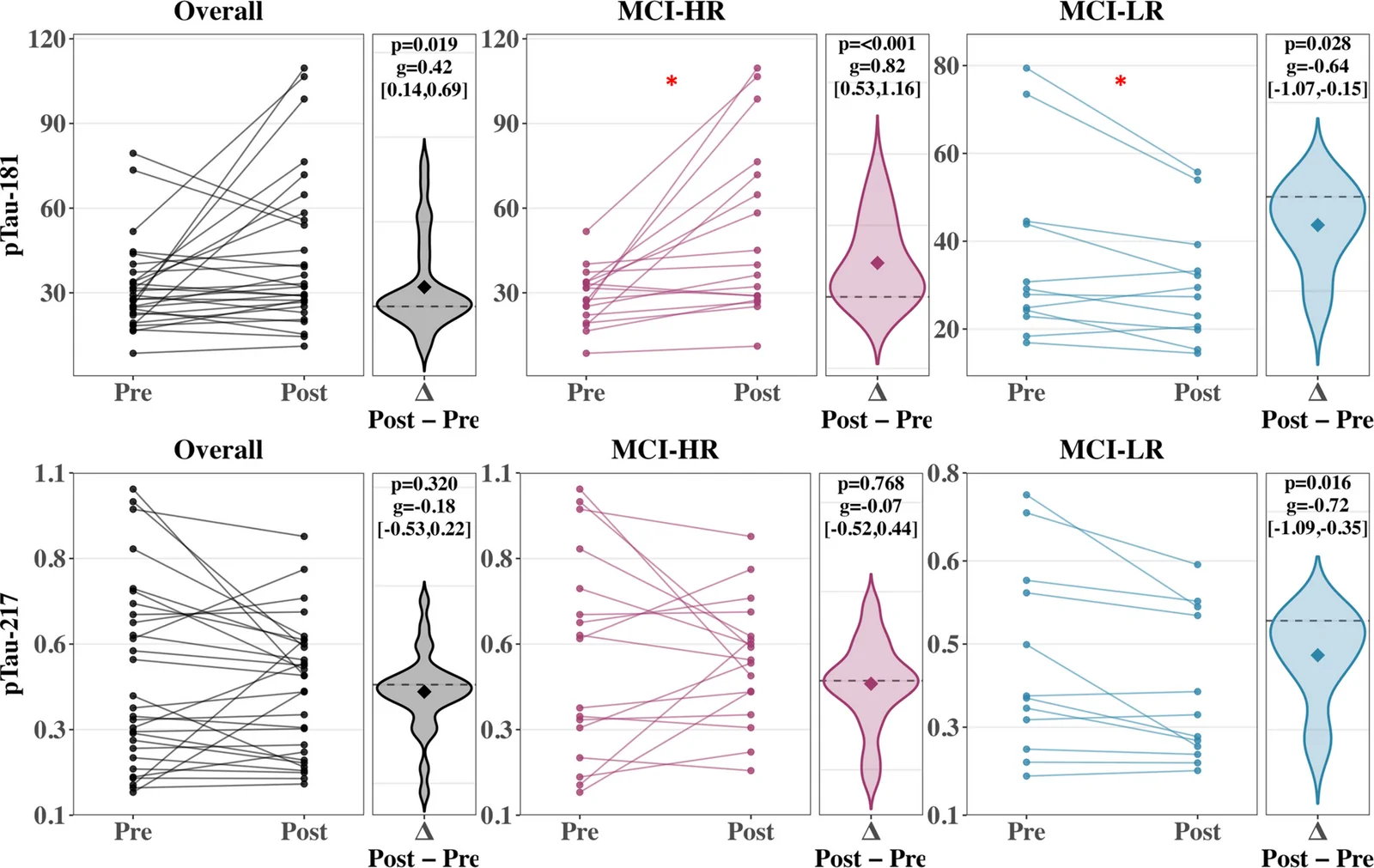
Pre–post changes in plasma p-tau181 and p-tau217. Pre–post changes in plasma pTau-181 (upper panels) and pTau-217 (lower panels) following oculomotor training, shown for the full sample (overall) and stratified by cognitive risk stratum (MCI-HR; MCI-LR). Left panels display individual trajectories (pre to post) and violin plots of the distribution of differences (Δ Post − Pre). In MCI-HR, pTau-181 increased significantly following the intervention, whereas in MCI-LR both pTau-181 and pTau-217 showed significant reductions. No between-stratum divergence reached significance for pTau-217. Values in pg/ml. Hedges’ *g* and 95% BCa bootstrap confidence intervals are shown within each panel; red asterisks indicate statistically significant between-group differences

Figure 1 illustrates pre–post changes in plasma p-tau181 and p-tau217 levels, showing divergent trajectories between MCI-LR and MCI-HR for p-tau181, and selective reductions in p-tau217 among MCI-LR.

### Effects of oculomotor training on cognition

Cognitive performance was analyzed using Freedman-Lane permutation tests with medication as a covariate, both in the full sample and stratified by cognitive state (MCI-LR and MCI-HR). Outcomes included the global MoCA score and the MoCA-MIS score.

**Fig. 2:**
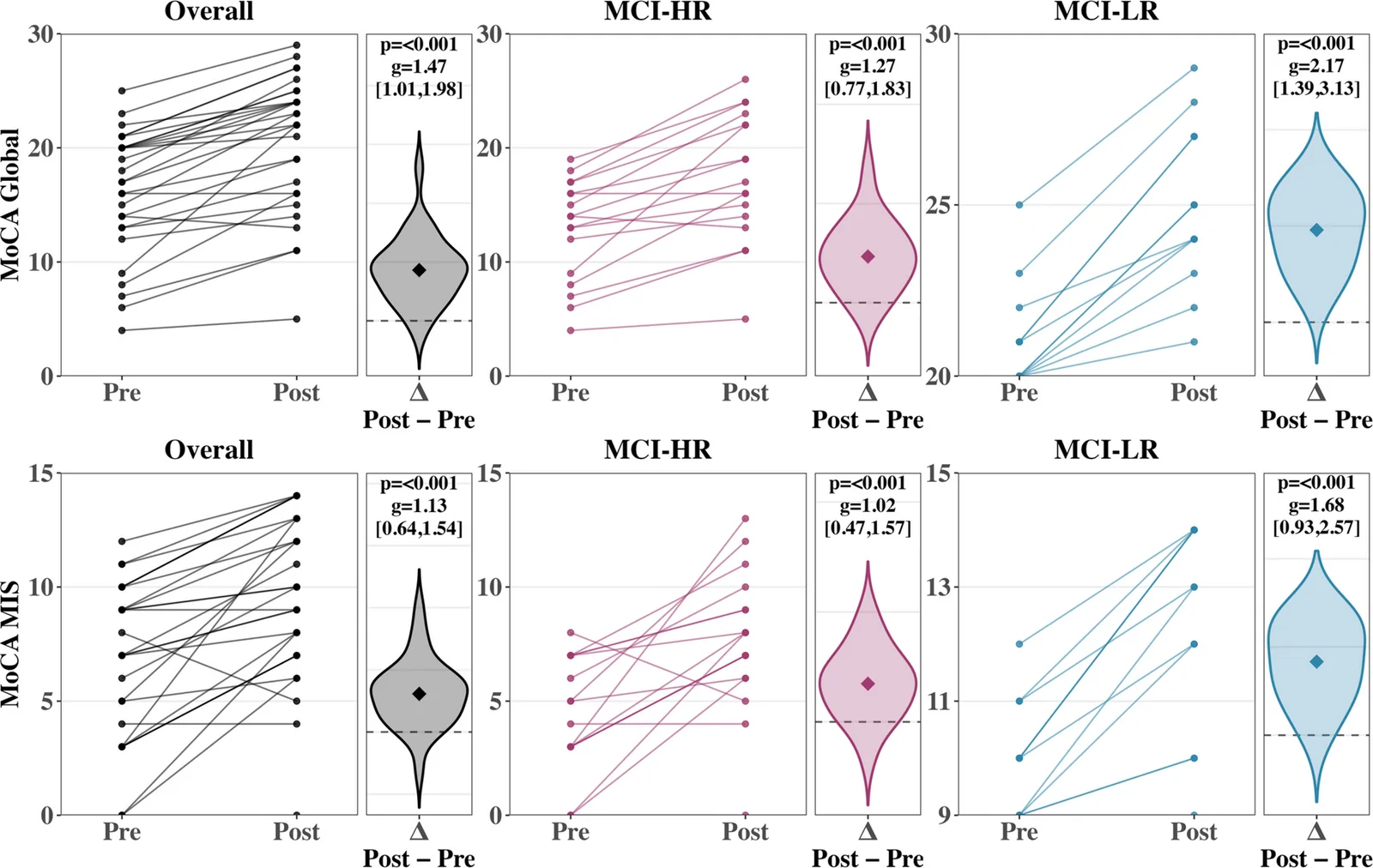
Pre–post changes in global cognition and memory performance. Pre–post changes in global cognitive performance (MoCA total score; upper panels) and memory-specific performance (MoCA-MIS; lower panels), shown for the full sample (overall) and stratified by cognitive risk stratum (MCI-HR; MCI-LR). Both groups showed significant improvements in MoCA total score and MoCA-MIS following the intervention, with no significant between-group difference in the magnitude of improvement. Left panels display individual trajectories and violin plots of the distribution of changes (Δ Post − Pre). Values expressed as MoCA score points. Hedges’ *g* and 95% BCa bootstrap confidence intervals are shown within each panel

Significant pre–post improvements were observed in both MoCA total score and MoCA-MIS across the full sample, with gains present in both subgroups and no significant between-group differences in the magnitude of improvement (MoCA Global: Hedges’ *g* = 0.28, 95% CI [−0.44, 0.90], *p* = 0.560; MoCA-MIS: Hedges’ *g* = 0.36, 95% CI [−0.36, 1.02], *p* = 0.304). Figure 2 illustrates pre–post changes in global cognition (MoCA total score) and memory performance (MoCA-MIS), for the full sample and stratified by cognitive status.

### Effects of oculomotor training on ocular signature

**Fig. 3:**
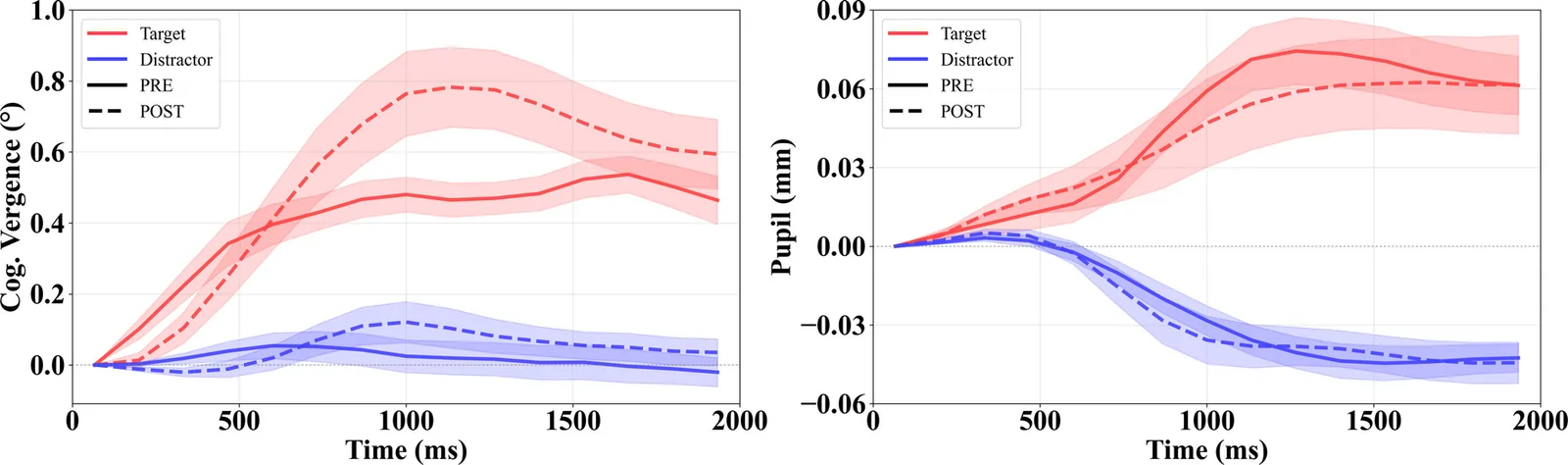
Cognitive vergence and pupil main curves. Grand-average cognitive vergence (left panel) and pupil diameter (right panel) time courses during the oddball task, pooled across all participants (*n* = 29) and assessment timepoints. Solid lines represent target trials (red) and distractor trials (blue); dashed lines represent post-intervention recordings. Shaded regions indicate 95% CI. Vergence is expressed in degrees (°) and pupil diameter in millimeters (mm). Target trials elicited consistently larger vergence and pupillary responses than distractor trials at both timepoints

All included participants (*n* = 29) completed the Oddball task before and after oculomotor training. Figure 3 shows the average pre- and post-training response of all participants in cognitive vergence and pupillary response.

#### Oculomotor signature of attentional processing

**Fig. 4:**
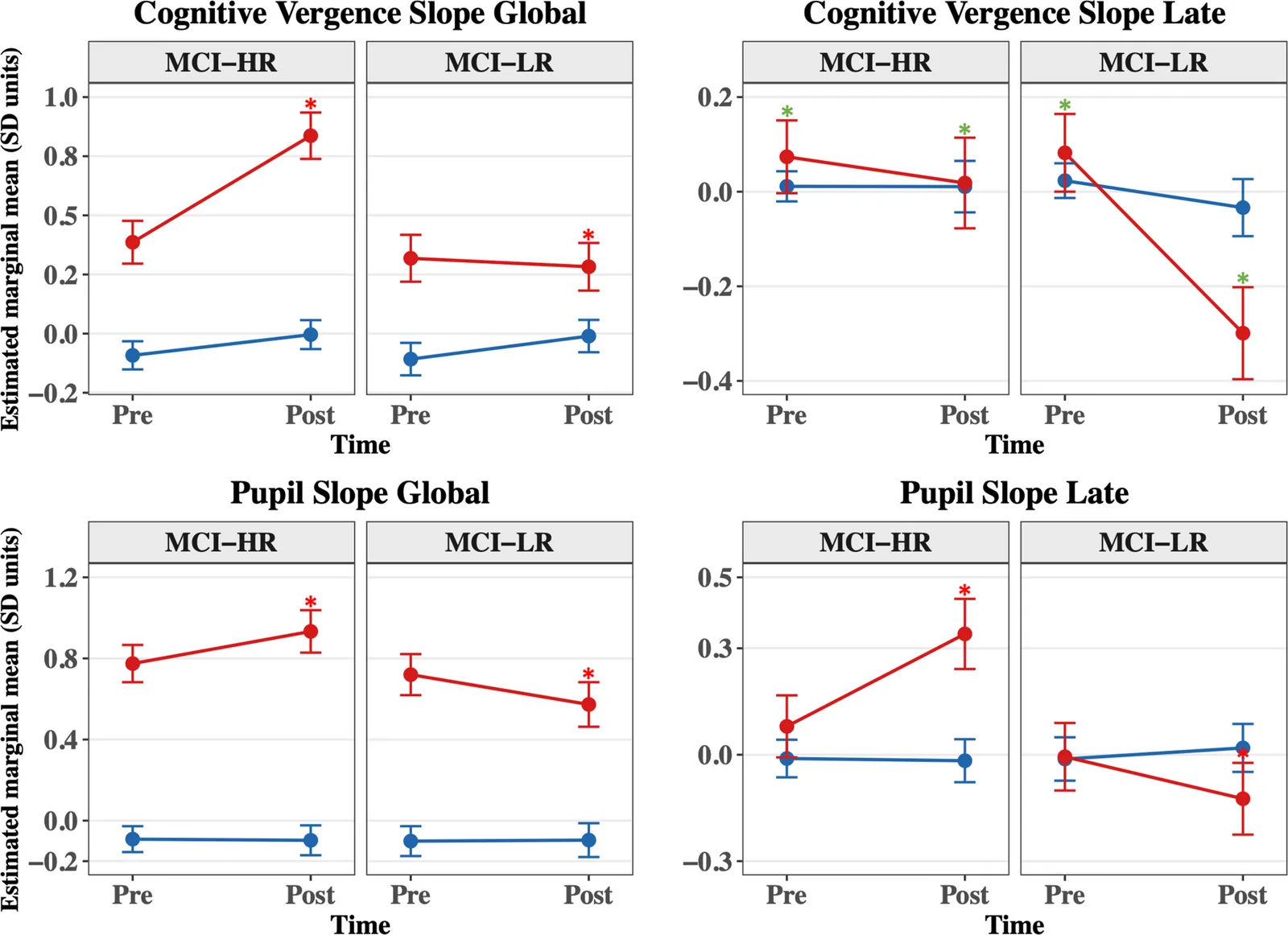
Estimated marginal means of cognitive vergence and pupil slope features before and after oculomotor training by cognitive state and stimulus type. Estimated marginal means and standard error derived from linear mixed-effects models for cognitive vergence slope (global and late) and pupil slope (global and late) across pre and post assessment timepoints, stratified by cognitive state (MCI-LR, MCI-HR) and stimulus type. Red lines indicate Target stimuli; blue lines indicate Distractor stimuli. Asterisks denote significant interactions: a red asterisk indicates a significant time × stimulus × cognitive state interaction (*p* < 0.05); a green asterisk indicates a significant time × stimulus interaction (*p* < 0.05). SD, standard deviation. Only significant results were found in target stimulus

Oculomotor signatures revealed selective and stimulus-dependent modulation following oculomotor training. All fixed-effects coefficients for slope features reflect standardized units (SD), as variables were z-scored prior to model fitting. For the global vergence slope, a significant three-way interaction (*β* = 0.496, 95% CI [0.124, 1.037], *p* = 0.004) indicated a selectively greater post-intervention increase for target stimuli in MCI-HR relative to MCI-LR. For the late vergence slope, a significant time × stimulus interaction (*β* = −0.324, 95% CI [−0.735, −0.041], *p* = 0.008) reflected a post-intervention reduction in sustained target-related vergence across both groups; although the triple interaction did not reach significance, estimated marginal means suggested this reduction was more pronounced in MCI-LR, while MCI-HR showed a numerically attenuated pattern. Pupil-based measures replicated this dissociation, with significant three-way interactions for both the global (*β* = 0.316, 95% CI [0.111, 1.013], *p* = 0.045) and late pupil slope (*β* = 0.417, 95% CI [0.054, 1.017], *p* = 0.013), reflecting divergent post-intervention trajectories whereby MCI-HR showed a greater increase in pupillary dilation relative to MCI-LR. No significant time-related modulation was observed for distractor trials or for remaining features. Complete fixed-effects estimates are reported in the Supplementary Material (Table S6) (Fig. 4).

### Behavioral performance during the oddball task

Accuracy was examined as a functional framework for oculomotor interpretation. Unlike oculomotor slope features, accuracy and reaction time coefficients are reported in their original units (proportion and milliseconds, respectively) to preserve functional interpretability. A significant main effect of Stimulus confirmed lower accuracy for target relative to distractor trials (*β* = −0.061, *p* < 0.001), consistent with the attentional demands of the oddball paradigm.

No significant three-way interaction was observed between time, stimulus, and diagnostic group for accuracy (*β* = −0.0274, SE = 0.019, *p* = 0.141). Post hoc analysis of estimated marginal means nonetheless revealed a pattern consistent with differential group responsiveness: MCI-HR participants showed a significant reduction in target accuracy following the intervention (estimate = −0.059, SE = 0.018, *p* = 0.002), whereas MCI-LR participants showed no significant change (estimate = −0.026, SE = 0.020, *p* = 0.197). No significant pre–post changes were observed in distractor accuracy in either group (MCI-HR: *p* = 0.408; MCI-LR: *p* = 0.731).

**Fig. 5:**
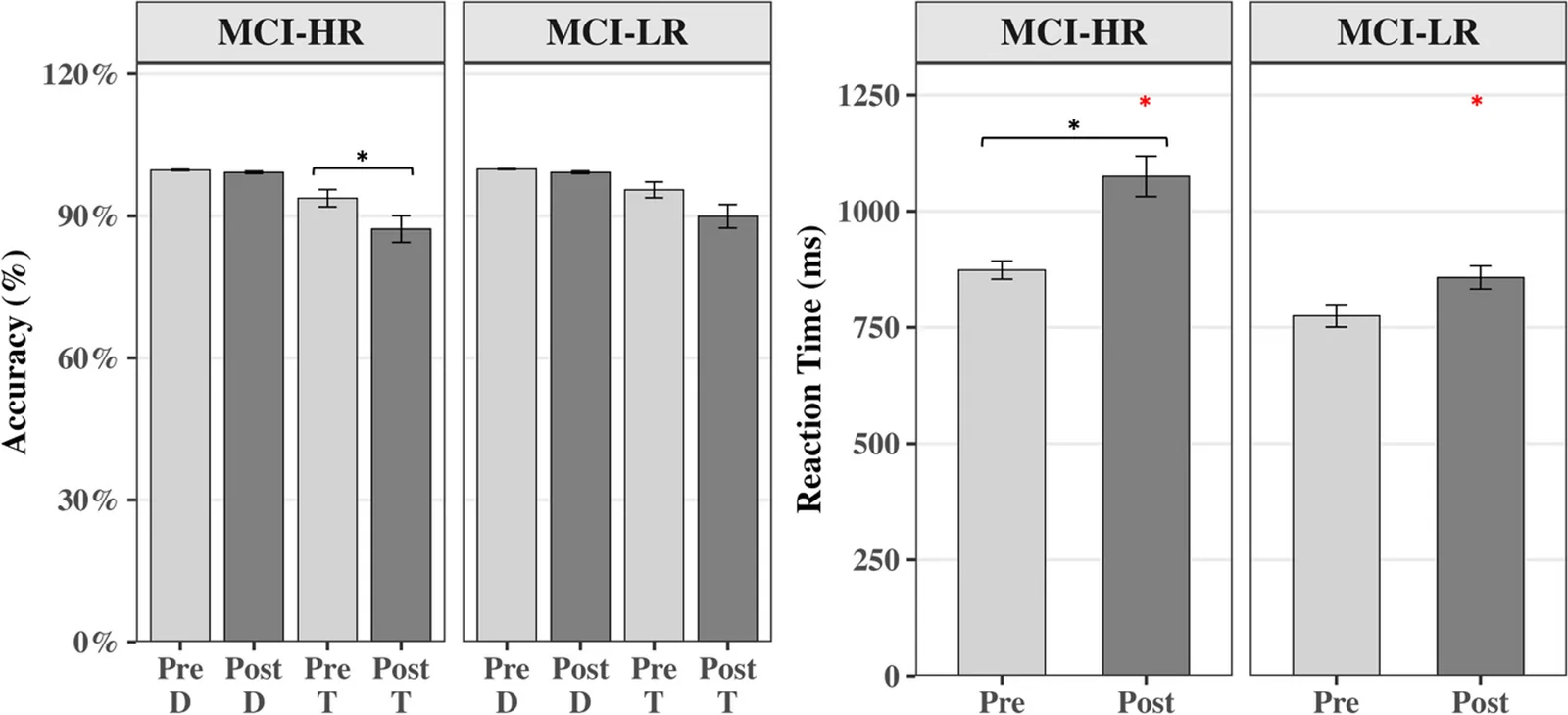
Behavioral response of attentional processing during the oddball task. Behavioral performance during the oddball task (accuracy and reaction time) at pre- and post-training timepoints, stratified by cognitive risk stratum (MCI-HR, MCI-LR) and trial type (Distractor, Target). MCI-HR participants showed a significant reduction in target accuracy and a significant increase in reaction time following the intervention, while MCI-LR participants maintained stable performance in both measures. Pre D, pre distractor; Post D, post distractor; Pre T, pre target; Post T, post target. The black asterisk indicates significant pre-to-post differences from post hoc contrasts; the red asterisk indicates significant between-group differences from mixed-model interactions

Reaction time for correct target trials showed a significant time × diagnostic group interaction (*β* = +196.4 ms, SE = 45.5, *p* < 0.001), indicating that MCI-HR participants became significantly slower following the intervention (estimate = +243.6 ms, SE = 32.4, *p* < 0.001), while MCI-LR participants maintained stable response speed (estimate = +47.2 ms, SE = 31.9, *p* = 0.140). Within MCI-HR, the co-occurrence of a significant reduction in target accuracy (*p* = 0.002) and a significant increase in reaction time (*p* < 0.001) is inconsistent with a speed-accuracy tradeoff, as both dimensions deteriorated simultaneously, indicating a global reduction in attentional processing efficiency. This pattern was absent in MCI-LR, where neither accuracy nor reaction time showed significant post-intervention changes, although the between-group divergence did not reach formal significance in the triple interaction. Figure 5 shows pre–post differences by condition and cognitive state.

### Integrative analyses across blood-based, cognitive, and oculomotor signature

**Fig. 6:**
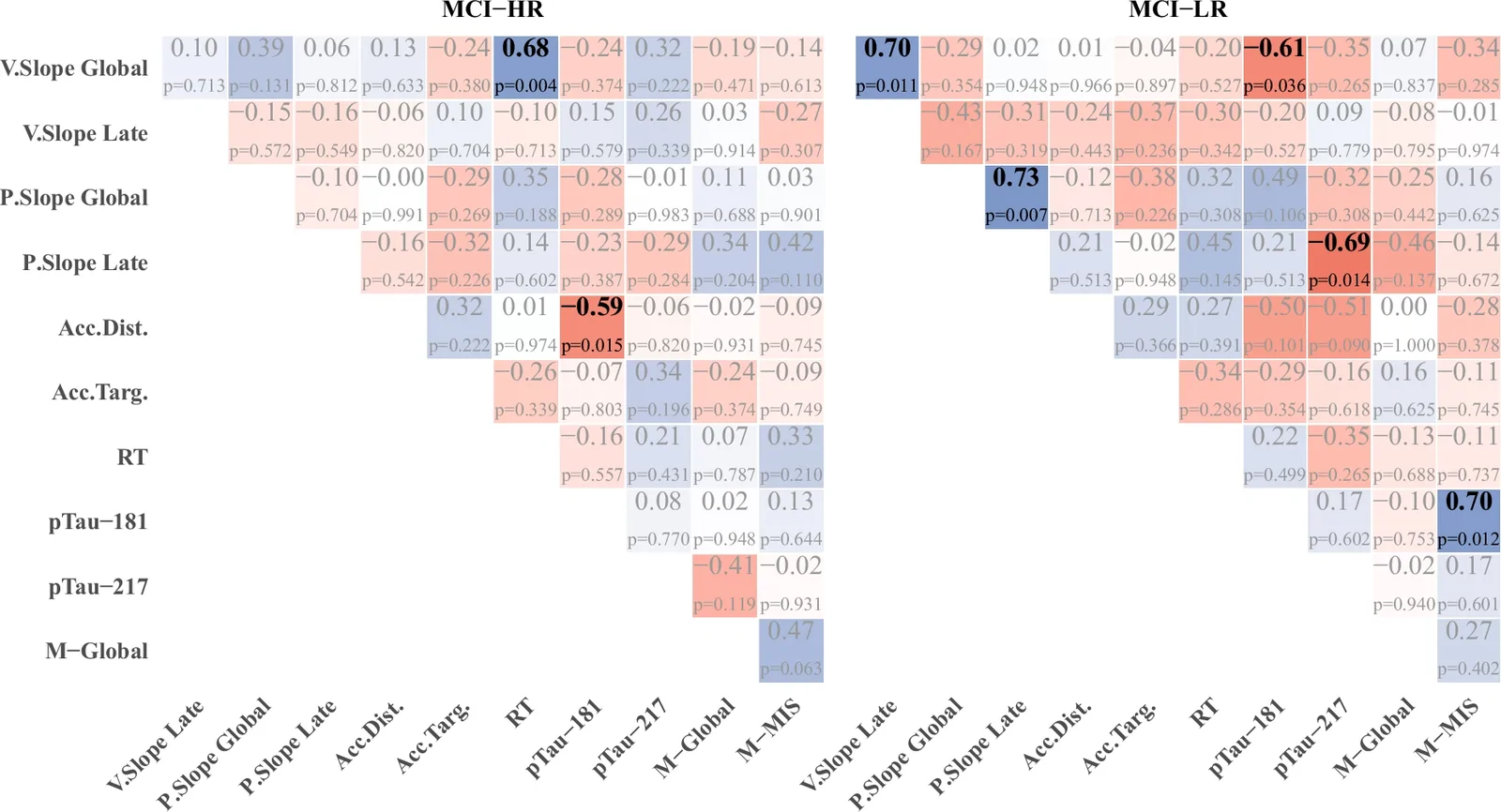
Spearman correlation heatmap between pre-to-post BLUPs of oculomotor features, behavioral measures, and biomarker/cognitive residuals. Each cell represents the Spearman correlation coefficient (*ρ*) between individual pre-to-post changes (BLUPs) derived from mixed-effects models. Cell color indicates the direction and magnitude of the correlation (blue: positive; red: negative). The coefficient is displayed in bold when statistically significant (*p* < 0.05), accompanied by the exact *p*-value. Only features with at least one significant interaction in the mixed models were included as well as significant pre–post changes in blood-based biomarkers and cognition. M-Global, MoCA Global Score; M-MIS, MoCA-MIS; RT, reaction time; Acc.Targ, accuracy target; Acc. Dist, accuracy distractor. P/V before feature indicates pupil or vergences respectably

Figure 6 shows pairwise Spearman correlations between individual pre-to-post changes across oculomotor, behavioral, and rank-residualized biological and cognitive variables, stratified by diagnostic group. The following analyses are exploratory and hypothesis-generating, given the small subgroup sizes (MCI-LR: *n* = 12; MCI-HR: *n* = 17) and the number of pairwise comparisons examined; they should not be interpreted as confirmatory evidence of mechanism.

In MCI-LR, significant within-domain coherence was observed between vergence and pupillary changes and between MoCA subscales. Three significant cross-domain associations were observed. Greater individual increases in vergence global slope were associated with greater relative reductions in pTau-181 (*ρ* = −0.60, 95% CI [−0.96, −0.20], *p* = 0.036). Greater individual increases in late pupil slope were associated with greater relative reductions in pTau-217 (*ρ* = −0.69, 95% CI [−0.95, −0.20], *p* = 0.014). Additionally, greater relative improvements in memory performance as indexed by MoCA-MIS were associated with greater relative increases in pTau-181 (*ρ* = 0.69, 95% CI [0.18, 0.79], *p* = 0.012). In MCI-HR, two significant cross-domain associations were observed: a positive relationship between vergence global slope change and reaction time change (*ρ* = 0.67, 95% CI [0.15, 0.90], *p* = 0.004), and an inverse relationship between distractor accuracy change and rank-residualized pTau-181 change (*ρ* = −0.59, 95% CI [−0.84, −0.14], *p* = 0.015). No other cross-domain correlations reached statistical significance in either group.

### Clinician-rated observational outcomes

Table 2 presents the descriptive distribution of clinician-rated scores across the five observational items for the full sample and stratified by diagnostic group. These data reflect the independent, blind assessments of two psychologists following 8 weeks of intervention and are presented as exploratory observational evidence; given the non-validated nature of the instrument, results should be interpreted with caution (see “Limitations”).

**Table 2:** Clinician-rated observational outcomes following oculomotor training

Group	Item	Median [IQR]	1 (no change)	2	3	4	5 (very notable change)
Full sample	Motivation and participation	3 [2,4]	16%	24%	28%	32%	0%
	Attention and concentration	2 [2,3]	20%	32%	48%	0%	0%
	Mood and well-being	2 [2,3]	16%	36%	32%	16%	0%
	Memory and daily orientation	2 [1,3]	36%	28%	36%	0%	0%
	Social interaction and initiative	2 [2,3]	8%	44%	40%	8%	0%
MCI-HR	Motivation and participation	4 [3,4]	7.7%	15.4%	23.1%	53.8%	0%
	Attention and concentration	3 [3]	7.7%	15.4%	76.9%	0%	0%
	Mood and well-being	3 [2,3]	0%	38.5%	46.2%	15.4%	0%
	Memory and daily orientation	3 [2,3]	15.4%	23.1%	61.5%	0%	0%
	Social interaction and initiative	3 [2,3]	0%	38.5%	46.2%	15.4%	0%
MCI-LR	Motivation and participation	2 [1.75, 3]	25%	33.3%	33.3%	8.3%	0%
	Attention and concentration	2 [1,2]	33.3%	50%	16.7%	0%	0%
	Mood and well-being	2 [1,3]	33.3%	33.3%	16.7%	16.7%	0%
	Memory and daily orientation	1 [1,2]	58.3%	33.3%	8.3%	0%	0%
	Social interaction and initiative	2 [2,3]	16.7%	50%	33.3%	0%	0%

Scale: 1 = no perceived change; 5 = very notable perceived change. This is a non-validated observational instrument; results are presented as exploratory descriptive evidence only and should be interpreted with caution (see “Limitations”)

*IQR*, interquartile range

Both psychologists reported perceived changes across all five domains, with the highest ratings for motivation and participation (Mdn = 3, IQR [2, 4]), suggesting potential ecological relevance warranting investigation with validated measures in future studies.

## Discussion

This study aimed to determine whether attentionally directed oculomotor training, administered through a gamified eye-tracking task, is associated with differential effects on blood-based neurodegeneration biomarkers, cognitive performance, and oculomotor signatures in individuals with MCI at differential risk of conversion to Alzheimer’s dementia. The results reveal a dissociation across molecular, cognitive, and oculomotor domains: whereas cognitive improvements were comparable between strata, plasma phosphorylated tau biomarkers followed divergent trajectories, with reductions in both p-tau181 and p-tau217 in MCI-LR and a significant increase in p-tau181 in MCI-HR, a divergence that was statistically significant between groups.

### Plasma biomarker and cognitive outcomes

Regarding plasma biomarkers, the reductions in both p-tau181 and p-tau217 in MCI-LR are compatible with, though do not demonstrate, the engagement of clearance-related mechanisms proposed in gamma stimulation models. The attentional demands of the gamified oculomotor task are hypothesized to engage LC-oculomotor circuits through frontal eye field and superior colliculus recruitment [20, 21], and phasic LC activation at gamma frequencies has been shown to drive cortical gamma oscillations alongside pupillary dilation [23, 26], providing a mechanistic link between attentionally directed oculomotor engagement and the gamma-related pathways implicated in glymphatic clearance. Murdock et al. (2024) demonstrated that multisensory 40 Hz stimulation promotes glymphatic clearance through vasoactive intestinal peptide interneuron activation and increased cerebrospinal fluid flow [13], and Milà-Alomà et al. (2022) established that p-tau217 shows greater sensitivity to early amyloid-associated tau phosphorylation, while p-tau181 reflects a larger dynamic soluble pool with greater inter-individual variability [34]. The parallel reduction across both tau species in MCI-LR is compatible with an intervention-associated modification of tau phosphorylation dynamics consistent with this mechanistic framework hypothesis.

The p-tau181 increase in MCI-HR warrants careful interpretation. The present design does not include a control group to establish whether the observed increase exceeds any natural trajectory in this stratum. That said, the concurrent stability of NfL, GFAP, amyloid markers, and p-tau217 is consistent with the interpretation that this increase does not reflect active neurodegeneration or accelerated tauopathy: NfL and GFAP elevation would be expected as early neurotoxicity indicators [42], and p-tau217 stability is inconsistent with accelerated tangle accumulation [34]. A possible interpretation, consistent with existing evidence on glymphatic dysfunction in AD [18, 19] and parvalbumin interneuron vulnerability in more advanced disease [16], is that oculomotor engagement may have partially activated gamma-related pathways sufficient to mobilize soluble tau into plasma, but in a context where reduced glymphatic clearance capacity limits subsequent elimination; the comparable cognitive gains in MCI-HR further argue against an adverse effect of the intervention. Nonetheless, these are indirect arguments, and the prior pilot study using the same training paradigm did not show significant changes in the control condition [27], providing partial contextual support that the divergent biomarker trajectories observed here are not attributable solely to non-specific effects, though a randomized controlled design with biological staging remains necessary to confirm this interpretation.

Regarding cognitive performance, improvements in MoCA and MoCA-MIS were comparable across both strata, consistent with prior reports of gamma sensory stimulation benefits in MCI populations [14, 15]. The absence of a randomized control group precludes complete exclusion of practice effects or examiner familiarity as contributing factors. Regression to the mean is an unlikely explanation for the observed MoCA gains: under regression to the mean, groups selected at opposite extremes of the baseline distribution would be expected to shift in opposite directions toward the population mean, whereas both MCI-HR and MCI-LR showed post-intervention improvement. This bidirectional improvement pattern is inconsistent with a regression-to-the-mean account [43]. Furthermore, the only available comparator, a pilot study employing the same paradigm with an active control group, observed no cognitive improvements in that arm [27], which reduces, without eliminating, the likelihood that the gains reported here reflect practice effects or examiner familiarity with the instrument rather than a genuine training response. Attentionally directed oculomotor training recruits vergence, saccadic, and antisaccadic movements through top-down demands, engaging circuits in the frontal eye fields, superior colliculus, and posterior parietal cortex implicated in attentional and executive processing [5, 20, 21]; the cognitive gains may thus reflect genuine engagement of these circuits through the LC-gamma pathway.

MCI-HR showed a significant reduction in target accuracy (*p* = 0.002) and a significant increase in reaction time (*p* < 0.001) following the intervention, while MCI-LR maintained stable performance in both measures. The co-occurrence of declining accuracy and increasing latency within MCI-HR rules out a speed-accuracy trade-off [44]: participants in this stratum were simultaneously less accurate and slower, indicating a global reduction in attentional processing efficiency that was not observed in MCI-LR. This within-group pattern in MCI-HR, though not formally divergent from MCI-LR at the triple interaction level, is consistent with greater vulnerability in attentional control at more advanced disease stages [1, 2] and may reflect a context in which the attentional demands of the training exceeded the available processing capacity in this stratum. Under the mechanistic framework proposed here, compromised parvalbumin interneuron integrity and reduced LC-gamma circuit capacity in MCI-HR may limit the extent to which attentionally directed oculomotor engagement can improve the temporal efficiency of attentional circuit recruitment, resulting in increased physiological resource mobilization without a corresponding behavioral benefit.

### Oculomotor signatures and mechanistic interpretation

Prior work established that vergence and pupil responses during the oddball task are sensitive to p-tau burden [8] and discriminate A+T+ from A-T+ profiles at the participant level [9], with temporal dynamics being more discriminative than magnitude measures. The present findings extend this pattern to an intervention context: significant training-related interactions emerged in the global and late slopes of both vergence and pupil signals but not in magnitude metrics, confirming that the temporal organization of attentional network recruitment is the domain most sensitive to both pathological burden and intervention-induced change.

The anatomical basis for this sensitivity is well established: cognitive vergence is tightly coupled to covert attention through mechanisms engaging the superior colliculus, frontal eye fields, and posterior parietal cortex [7, 33, 45], while pupil dynamics serve as a proxy of LC phasic firing and noradrenergic arousal modulation [5, 7, 46]. In MCI-LR, the post-intervention reduction in pupillary global and late slopes is consistent with more efficient attentional network recruitment requiring lower cognitive resource mobilization [6, 47, 48] and aligns with the LC-gamma framework established above: if training recalibrated LC-oculomotor circuit engagement in this stratum, a reduction in the pupillary effort signature during target processing could reflect recalibrated LC-oculomotor circuit engagement; however, this interpretation remains speculative and was not directly tested and is offered alongside the concurrent biomarker reductions observed.

In MCI-HR, the co-occurrence of increased vergence, increased pupillary responses, reduced target accuracy, and increased reaction time indicates that greater oculomotor and autonomic resource mobilization did not translate into more effective attentional processing. This dissociation between physiological effort and behavioral outcome mirrors the biomarker pattern in this stratum and is consistent with fragmented neural compensation at more advanced disease stages, where compromised gamma-generating circuit integrity, reduced alpha deactivation, and the attenuation of compensatory cerebral hyperactivation [16, 48–50] may collectively limit the functional reorganization that oculomotor training could otherwise produce.

### Integrative correlation analyses

The stratified correlation analyses, treated here as exploratory given the small subgroup sizes, provide additional perspective on these qualitative differences. In MCI-LR, greater individual increases in vergence global slope were associated with greater relative reductions in p-tau181 (*ρ* = −0.60, 95% CI [−0.96, −0.20], *p* = 0.036), and greater increases in late pupil slope were associated with greater relative reductions in p-tau217 (*ρ* = −0.69, 95% CI [−0.95, −0.20], *p* = 0.014). These associations suggest that, in MCI-LR, greater individual oculomotor engagement co-varied with greater relative reductions in plasma tau, a pattern consistent with the hypothesis that individual differences in LC-attentional circuit integrity, indexed by oculomotor recruitment magnitude [5, 47], may relate to the capacity for gamma-associated glymphatic clearance [13, 17, 18]. The association between MoCA-MIS improvement and p-Tau181 change further suggests that LC engagement magnitude may jointly determine cognitive benefit and tau mobilization dynamics prior to glymphatic elimination [13, 51] although this cannot be established from correlational data alone. This pattern is mechanistically distinct from the p-Tau181 increase observed in MCI-HR, which may reflect tau mobilization without effective glymphatic clearance, as suggested by the concurrent stability of p-Tau217 in that group despite reductions in both tau species in MCI-LR [34, 51, 52], rather than from direct measurement of glymphatic function.

In MCI-HR, the absence of oculomotor-tau coupling, alongside the significant positive association between vergence slope change and reaction time deterioration and the inverse relationship between distractor accuracy and p-Tau181 change (see “Results,” Sections. “Integrative analyses across blood-based, cognitive, and oculomotor signature”), suggests a compartmentalization of intervention effects in this stratum. Greater relative oculomotor engagement was associated with worse behavioral outcomes rather than molecular change, consistent with the interpretation that compromised gamma-generating circuit integrity in MCI-HR limits the extent to which LC-oculomotor recruitment can produce the coordinated molecular and functional reorganization observed in MCI-LR [16, 48–50]. Notably, MoCA improvements in MCI-HR were comparable in magnitude to those in MCI-LR yet showed no significant correlations with oculomotor, behavioral, or biomarker changes, suggesting that the cognitive gains captured by global screening may operate through pathways at least partially independent of LC-attentional circuit integrity, possibly involving compensatory cortical recruitment accessible even when deeper attentional network reorganization is limited [49, 53].

### Clinical implications

The comparable cognitive improvements across both disease stages support the feasibility of oculomotor training as an accessible intervention in MCI regardless of conversion risk profile, with the short, gamified format compatible with home administration. The divergent biomarker trajectories highlight the importance of biological staging in gamma-based trials: the pTau-181 increase in MCI-HR underscores that plasma biomarker responses cannot be interpreted uniformly across the MCI spectrum, an interpretation supported by the concurrent stability of NfL, GFAP, and amyloid markers. The oculomotor-molecular coupling patterns suggest that vergence and pupil temporal dynamics may serve as functional indices of neuroplasticity capacity for patient stratification, with preserved coupling identifying individuals most likely to benefit molecularly, and compartmentalized responses identifying those requiring earlier or combined interventions. The clinician observations, with highest ratings for motivation and participation, suggest ecological relevance that future trials should formally assess using validated functional measures.

### Strengths and limitations

This study is, to our knowledge, the first to examine attentionally directed oculomotor training effects on blood-based neurodegeneration biomarkers, cognition, and oculomotor signatures within a single protocol in MCI. The plasma biomarker panel covering tau phosphorylation, amyloid metabolism, and neurodegeneration, combined with cognitive and oculomotor data, enables cross-domain analyses undetectable through isolated assessments. Robust small-sample inference was ensured through permutation-based testing, BCa bootstrap for effect estimation, and LOSO stability evaluation, collectively confirming that no single participant drove any reported effect and that all significant findings were stable across the full range of leave-one-out iterations. The short, gamified session format and high adherence support translational feasibility for clinical and home settings.

Several limitations must be acknowledged. The primary limitation is the absence of a randomized control group, which precludes definitive attribution of the observed effects to the intervention and does not allow exclusion of practice effects on cognitive measures, despite the evidence from the prior pilot study [27]. The single post-intervention timepoint does not allow determination of whether effects are sustained or whether the pTau-181 increase in MCI-HR represents a transient phenomenon. The significant sex imbalance between strata and the recruitment from residential care facilities limit generalizability to community-dwelling populations. Because participants were recruited from residential care facilities, data protection regulations restricted access to variables beyond the primary outcomes and eligibility criteria; consequently, somatic comorbidities such as vascular risk factors, diabetes, and psychiatric history could not be systematically documented, precluding a full assessment of baseline clinical comparability between strata. Sample size was calculated for the overall pre–post comparison; the MCI-HR (*n* = 17) and MCI-LR (*n* = 12) subgroup analyses therefore carry reduced statistical power relative to this calculation, yielding a lower signal-to-noise ratio for stratum-specific estimates. This constraint was partly addressed through permutation-based inference, BCa bootstrap confidence intervals, and leave-one-subject-out stability analysis (Table S1), which confirmed the absence of sign reversals or influential single-participant effects for all significant findings; nonetheless, reduced power limits the precision of stratum-specific estimates. Future studies incorporating AT(N) biological staging, randomized designs, and extended follow-up are needed to confirm these findings.

## Conclusion

This study provides preliminary evidence that attentionally directed oculomotor training produces cognitive improvements across the MCI spectrum while generating qualitatively distinct molecular, behavioral, and oculomotor signature responses depending on disease stage. In MCI-LR, the intervention was associated with reductions in both plasma pTau-181 and pTau-217, stable behavioral performance, and integrated oculomotor-molecular coupling patterns consistent with a coordinated neuroplasticity response mediated by preserved LC-attentional circuits and functional glymphatic clearance. In MCI-HR, comparable cognitive gains occurred alongside a pTau-181 increase consistent with stimulation-induced tau mobilization without effective glymphatic clearance, reduced target detection accuracy, and a compartmentalized oculomotor signature reflecting increased attentional effort rather than improved network efficiency. These findings suggest, with the caution warranted by the present sample size, the existence of a therapeutic window at the MCI-LR disease stage critically dependent on gamma-generating circuit integrity and glymphatic clearance capacity and support the development of patient stratification strategies based on disease stage and neuroplasticity biomarkers for future gamma stimulation clinical trials.

## Supplementary Information

Below is the link to the electronic supplementary material.

ESM 1 (DOCX 741 KB)

## Data Availability

Data are available upon reasonable request to the corresponding author. The method of cognitive vergence for neurodegeneration and measuring neurodegenerative proteins has been protected by a patent application.
