# Supplementary material for: Attentionally directed oculomotor training produces disease-stage-dependent effects on plasma tau, cognition, and oculomotor signatures in patients with mild cognitive impairment
Source: GeroScience. 2026 Aug 12;48(6):7523–44. doi: 10.1007/s11357-026-02474-7 (PMC13638860; doi:10.1007/s11357-026-02474-7)
Supplement: Supplementary file 1 [file 11357_2026_2474_MOESM1_ESM.docx]

Table S1. Leave-one-subject-out sensitivity analysis for all significant oculomotor effects

| **Feature** | **Term** | **β original** | **Mean β LOSO** | **Min β LOSO** | **Max β LOSO** | **N influential** |
| --- | --- | --- | --- | --- | --- | --- |
| Vergence slope (global) | Post × Target × MCI-AD | 0.496 | 0.496 | 0.357 | 0.545 | 0 |
| Vergence slope (late) | Post × Target | −0.324 | −0.324 | −0.397 | −0.195 | 0 |
| Pupil slope (global) | Post × Target × MCI-AD | 0.316 | 0.317 | 0.128 | 0.458 | 0 |
| Pupil slope (late) | Post × Target × MCI-AD | 0.416 | 0.416 | 0.238 | 0.508 | 0 |

Note. LOSO analysis was performed by sequentially excluding each of the 29 participants and refitting the full linear mixed-effects model on the remaining observations. original: fixed-effects estimate from the full-sample model. All four effects showed no sign reversals across any iteration and no influential observations were identified.

The sensitivity analysis including medication as a covariate was conducted using Wald-based inference, justified by the close correspondence between Wald and permutation-based p-values observed across all primary models, where both approaches identified the same significant effects.

Table S2. Sensitivity analysis full oculomotor signature response with medication.

| **Feature** | **Term** | **β** | **SE** | **p-value** |
| --- | --- | --- | --- | --- |
| **Vergence Slope Initial** | Stimulus (Target) | 0.418 | 0.085 | **<0.001** |
|  | Time (Post) | -0.117 | 0.061 | 0.068 |
|  | Medication (Yes) | -0.086 | 0.063 | 0.185 |
|  | Post × Target | -0.109 | 0.121 | 0.370 |
|  | Post × MCI-AD | 0.067 | 0.082 | 0.422 |
|  | Target × MCI-AD | -0.077 | 0.116 | 0.504 |
|  | Post × Target × MCI-AD | 0.078 | 0.169 | 0.644 |
| **Vergence Slope Global** | Stimulus (Target) | 0.427 | 0.083 | **<0.001** |
|  | Time (Post) | 0.097 | 0.080 | 0.234 |
|  | Medication (Yes) | -0.077 | 0.081 | 0.351 |
|  | Post × Target | -0.134 | 0.119 | 0.259 |
|  | Post × MCI-AD | -0.009 | 0.107 | 0.931 |
|  | Target × MCI-AD | 0.052 | 0.113 | 0.645 |
|  | Post × Target × MCI-AD | 0.496 | 0.166 | **0.003** |
| **Vergence Slope Late** | Stimulus (Target) | 0.058 | 0.085 | 0.497 |
|  | Time (Post) | -0.056 | 0.065 | 0.390 |
|  | Medication (Yes) | 0.055 | 0.048 | 0.263 |
|  | Post × Target | -0.323 | 0.122 | **0.008** |
|  | Post × MCI-AD | 0.056 | 0.087 | 0.522 |
|  | Target × MCI-AD | 0.002 | 0.117 | 0.985 |
|  | Post × Target × MCI-AD | 0.270 | 0.171 | 0.114 |
| **Vergence AUC** | Stimulus (Target) | 0.621 | 0.082 | **<0.001** |
|  | Time (Post) | 0.010 | 0.079 | 0.902 |
|  | Medication (Yes) | -0.148 | 0.089 | 0.109 |
|  | Post × Target | -0.051 | 0.117 | 0.663 |
|  | Post × MCI-AD | 0.056 | 0.105 | 0.602 |
|  | Target × MCI-AD | -0.009 | 0.112 | 0.937 |
|  | Post × Target × MCI-AD | 0.245 | 0.164 | 0.135 |
| **Vergence Peak Power** | Stimulus (Target) | -0.063 | 0.075 | 0.403 |
|  | Time (Post) | 0.014 | 0.191 | 0.940 |
|  | Medication (Yes) | -0.297 | 0.181 | 0.114 |
|  | Post × Target | 0.195 | 0.107 | 0.069 |
|  | Post × MCI-AD | 0.208 | 0.252 | 0.418 |
|  | Target × MCI-AD | 0.042 | 0.102 | 0.679 |
|  | Post × Target × MCI-AD | 0.133 | 0.149 | 0.373 |
| **Vergence Time to Peak** | Stimulus (Target) | 0.109 | 0.085 | 0.201 |
|  | Time (Post) | 0.036 | 0.088 | 0.687 |
|  | Medication (Yes) | 0.081 | 0.063 | 0.209 |
|  | Post × Target | -0.119 | 0.122 | 0.332 |
|  | Post × MCI-AD | -0.082 | 0.118 | 0.495 |
|  | Target × MCI-AD | 0.010 | 0.116 | 0.930 |
|  | Post × Target × MCI-AD | 0.249 | 0.170 | 0.144 |
| **Pupil Slope Initial** | Stimulus (Target) | 0.138 | 0.086 | 0.107 |
|  | Time (Post) | 0.066 | 0.069 | 0.347 |
|  | Medication (Yes) | -0.017 | 0.045 | 0.702 |
|  | Post × Target | -0.097 | 0.123 | 0.429 |
|  | Post × MCI-AD | -0.040 | 0.092 | 0.669 |
|  | Target × MCI-AD | 0.057 | 0.117 | 0.626 |
|  | Post × Target × MCI-AD | 0.069 | 0.171 | 0.687 |
| **Pupil Slope Global** | Stimulus (Target) | 0.822 | 0.080 | **<0.001** |
|  | Time (Post) | 0.005 | 0.067 | 0.944 |
|  | Medication (Yes) | -0.155 | 0.099 | 0.132 |
|  | Post × Target | -0.153 | 0.115 | 0.184 |
|  | Post × MCI-AD | -0.011 | 0.090 | 0.907 |
|  | Target × MCI-AD | 0.046 | 0.110 | 0.678 |
|  | Post × Target × MCI-AD | 0.316 | 0.161 | **0.011** |
| **Pupil Slope Late** | Stimulus (Target) | 0.007 | 0.085 | 0.938 |
|  | Time (Post) | 0.031 | 0.082 | 0.706 |
|  | Medication (Yes) | -0.053 | 0.073 | 0.476 |
|  | Post × Target | -0.150 | 0.121 | 0.215 |
|  | Post × MCI-AD | -0.037 | 0.109 | 0.734 |
|  | Target × MCI-AD | 0.085 | 0.116 | 0.465 |
|  | Post × Target × MCI-AD | 0.417 | 0.169 | **0.014** |
| **Pupil AUC** | Stimulus (Target) | 0.734 | 0.081 | **<0.001** |
|  | Time (Post) | 0.020 | 0.072 | 0.785 |
|  | Medication (Yes) | -0.117 | 0.096 | 0.233 |
|  | Post × Target | -0.140 | 0.116 | 0.230 |
|  | Post × MCI-AD | -0.040 | 0.096 | 0.679 |
|  | Target × MCI-AD | 0.040 | 0.111 | 0.716 |
|  | Post × Target × MCI-AD | 0.219 | 0.163 | 0.178 |
| **Pupil Peak Power** | Stimulus (Target) | 0.091 | 0.076 | 0.229 |
|  | Time (Post) | 0.116 | 0.150 | 0.447 |
|  | Medication (Yes) | 0.038 | 0.181 | 0.835 |
|  | Post × Target | -0.165 | 0.108 | 0.127 |
|  | Post × MCI-AD | 0.062 | 0.199 | 0.758 |
|  | Target × MCI-AD | 0.048 | 0.103 | 0.642 |
|  | Post × Target × MCI-AD | 0.267 | 0.151 | 0.078 |
| **Pupil Time to Peak** | Stimulus (Target) | 0.103 | 0.085 | 0.222 |
|  | Time (Post) | -0.032 | 0.116 | 0.786 |
|  | Medication (Yes) | 0.048 | 0.054 | 0.800 |
|  | Post × Target | 0.109 | 0.121 | 0.370 |
|  | Post × MCI-AD | -0.064 | 0.154 | 0.681 |
|  | Target × MCI-AD | -0.024 | 0.116 | 0.836 |
|  | Post × Target × MCI-AD | -0.195 | 0.170 | 0.251 |

Reference category: MCI-NC, Pre-intervention, Distractor trials, no medication. Coefficients are standardized and represent effect sizes in standard deviation units. Positive coefficients indicate increases relative to reference. Interaction terms represent differences in effects between conditions. Medication (Yes) was included as a covariate to control for the potential confounding effect of cholinergic and anticholinergic drug use. All models included random intercepts and random slopes for time by participant. In bold significant p-values.

Table S3. Sensitivity analysis: sex as covariate in oculomotor mixed models.

| **Feature** | **Term** | **β** | **SE** | **p-value** |
| --- | --- | --- | --- | --- |
| **Vergence Slope Global** | Sex (Female) | 0.085 | 0.084 | 0.317 |
|  | Post × MCI-AD | -0.009 | 0.107 | 0.933 |
|  | Post × Target | -0.134 | 0.119 | 0.258 |
|  | Post × Target × MCI-AD | 0.497 | 0.166 | **0.003** |
| **Vergence Slope Late** | Sex (Female) | -0.044 | 0.050 | 0.380 |
|  | Post × MCI-AD | 0.056 | 0.086 | 0.522 |
|  | Post × Target | -0.324 | 0.122 | **0.008** |
|  | Post × Target × MCI-AD | 0.269 | 0.171 | 0.115 |
| **Pupil Slope Global** | Sex (Female) | -0.014 | 0.108 | 0.900 |
|  | Post × MCI-AD | -0.011 | 0.090 | 0.905 |
|  | Post × Target | -0.152 | 0.115 | 0.186 |
|  | Post × Target × MCI-AD | 0.317 | 0.161 | **0.049** |
| **Pupil Slope Late** | Sex (Female) | 0.107 | 0.073 | 0.153 |
|  | Post × MCI-AD | -0.037 | 0.109 | 0.739 |
|  | Post × Target | -0.152 | 0.121 | 0.211 |
|  | Post × Target × MCI-AD | 0.417 | 0.169 | **0.014** |

Reference category: MCI-NC, Pre-intervention, Distractor trials, Male. Coefficients are standardized and represent effect sizes in standard deviation units. Sex (Female) was added as an additive covariate to the full Diagcat × Time × Stimulus mixed-effects model to assess its influence on the three primary interaction terms. Inference is Wald-based (REML), without permutation or bootstrap. All models included random intercepts and random slopes for time by participant. In bold, significant p-values.

Table S4. Sensitivity analysis: sex as covariate in biomarker and cognitive Freedman-Lane permutation models.

| **Biomarker** | **Group** | **p-value (original)** | **p-value (+Sex)** |
| --- | --- | --- | --- |
| **pTau-181** | Overall | **0.0195** | **0.0201** |
|  | MCI-HR | **<0.001** | **<0.001** |
|  | MCI-LR | **0.0283** | **0.0311** |
|  | Between-group | **0.0014** | **0.0053** |
| **pTau-217** | Overall | 0.3204 | 0.3249 |
|  | MCI-HR | 0.7679 | 0.7761 |
|  | MCI-LR | **0.0158** | **0.0147** |
|  | Between-group | 0.6314 | 0.9734 |
| **MoCA Global** | Overall | **<0.001** | **<0.001** |
|  | MCI-HR | **<0.001** | **<0.001** |
|  | MCI-LR | **<0.001** | **<0.001** |
|  | Between-group | 0.5598 | 0.3682 |
| **MoCA MIS** | Overall | **<0.001** | **<0.001** |
|  | MCI-HR | **<0.001** | **<0.001** |
|  | MCI-LR | **<0.001** | **<0.001** |
|  | Between-group | 0.3035 | 0.1457 |

p-value (original) corresponds to the Freedman-Lane permutation model as reported in the main text (10,000 permutations, no covariate for these outcomes). p-value (+Sex) corresponds to the same permutation procedure with sex added as a covariate. Between-group refers to the MCI-HR vs. MCI-LR comparison of pre-to-post change. No effect changed significance status (α = 0.05) after adjusting for sex. In bold, significant p-values.

Table S5. Biomarker Summary Table

|  | **Overall** |  | | **MCI-HR** |  | | **MCI-LR** |  | | **Between-group** |  |
| --- | --- | --- | --- | --- | --- | --- | --- | --- | --- | --- | --- |
| **Biomarker** | **M ± SD** | **g [95%CI]** | **p** | **M ± SD** | **g [95%CI]** | **p** | **M ± SD** | **g [95%CI]** | **p** | **g [95%CI]** | **p** |
| **Aβ 40** | 148.64 ± 61.28 | 0.11 [-0.27,  0.47] | 0.548 | 132.95 ± 32.54 | 0.35 [-0.14,  0.85] | 0.127 | 170.87 ± 84.26 | -0.25 [-0.77, 0.32] | 0.382 | 0.62 [-0.12,  1.34] | 0.090 |
| **Aβ 42** | 8.14 ± 3.07 | -0.09 [-0.40, 0.31] | 0.671 | 7.41 ± 2.31 | 0.22 [-0.30,  0.68] | 0.358 | 9.18 ± 3.78 | -0.35 [-0.69, 0.33] | 0.213 | 0.63 [-0.14,  1.20] | 0.108 |
| **Ratio  42/40** | 0.06 ± 0.01 | -0.19 [-0.50, 0.21] | 0.317 | 0.06 ± 0.01 | -0.03 [-0.55, 0.46] | 0.895 | 0.06 ± 0.01 | -0.35 [-0.66, 0.28] | 0.183 | 0.39 [-0.37,  1.00] | 0.365 |
| **GFAP** | 109.23 ± 47.37 | 0.13 [-0.27, 0.42] | 0.554 | 119.75 ± 53.81 | 0.17 [-0.39,  0.52] | 0.576 | 94.32 ± 32.95 | 0.03 [-0.55,  0.62] | 0.915 | 0.20 [-0.56,  0.72] | 0.623 |
| **NfL** | 28.97 ± 17.40 | -0.15 [-0.41, 0.24] | 0.506 | 27.90 ± 16.16 | -0.32 [-0.58, 0.24] | 0.140 | 30.49 ± 19.67 | 0.34 [-0.29,  1.03] | 0.239 | -0.59 [-1.03, 0.18] | 0.084 |

Values represent pre-intervention means ± standard deviation. g = Hedges' g effect size; 95% CI = bias-corrected and accelerated bootstrap confidence interval (10,000 iterations). Pre-to-post comparisons within each group were tested using Freedman-Lane permutation tests (10,000 permutations) with medication use as a covariate, permuting residuals of the reduced model within participants. Between-group comparisons reflect independent-samples Hedges' g computed on individual pre-to-post delta scores (MCI-HR minus MCI-LR), tested via Freedman-Lane permutation on a linear model of deltas regressed on group and medication. A negative g in pre-to-post comparisons indicates post-intervention reduction; a positive g indicates increase. A positive between-group g indicates a larger increase (or smaller reduction) in MCI-HR relative to MCI-LR. Aβ = amyloid-beta; GFAP = glial fibrillary acidic protein; NfL = neurofilament light chain.

Table S6. Full oculomotor signature response

| **Feature** | **Term** | **β** | **95% CI** | **p-value** |
| --- | --- | --- | --- | --- |
| **Vergence Slope Initial** | Stimulus (Target) | 0.417 | 0.250, 0.584 | **<0.001** |
|  | Time (Post) | -0.117 | -0.237, 0.003 | 0.068 |
|  | Post × Target | -0.107 | -0.408, 0.220 | 0.408 |
|  | Post × MCI-HR | 0.066 | -0.074, 0.306 | 0.427 |
|  | Target × MCI-HR | -0.078 | -0.305, 0.149 | 0.501 |
|  | Post × Target × MCI-HR | 0.077 | -0.336, 0.465 | 0.668 |
| **Vergence Slope Global** | Stimulus (Target) | 0.426 | 0.263, 0.589 | **<0.001** |
|  | Time (Post) | 0.098 | -0.059, 0.255 | 0.234 |
|  | Post × Target | -0.133 | -0.263, 0.004 | 0.252 |
|  | Post × MCI-HR | -0.010 | -0.180, 0.203 | 0.889 |
|  | Target × MCI-HR | 0.052 | -0.169, 0.273 | 0.647 |
|  | Post × Target × MCI-HR | 0.496 | 0.124, 1.037 | **0.004** |
| **Vergence Slope Late** | Stimulus (Target) | 0.059 | -0.107, 0.225 | 0.490 |
|  | Time (Post) | -0.057 | -0.183, 0.069 | 0.384 |
|  | Post × Target | -0.324 | -0.735, -0.041 | **0.008** |
|  | Post × MCI-HR | 0.056 | -0.095, 0.204 | 0.526 |
|  | Target × MCI-HR | 0.004 | -0.223, 0.231 | 0.975 |
|  | Post × Target × MCI-HR | 0.270 | -0.130, 0.779 | 0.097 |
| **Vergence AUC** | Stimulus (Target) | 0.620 | 0.459, 0.781 | **<0.001** |
|  | Time (Post) | 0.010 | -0.145, 0.165 | 0.900 |
|  | Post × Target | -0.050 | -0.359, 0.325 | 0.675 |
|  | Post × MCI-HR | 0.055 | -0.124, 0.307 | 0.561 |
|  | Target × MCI-HR | -0.010 | -0.229, 0.209 | 0.932 |
|  | Post × Target × MCI-HR | 0.245 | -0.185, 0.662 | 0.131 |
| **Vergence Peak Power** | Stimulus (Target) | -0.063 | -0.210, 0.084 | 0.400 |
|  | Time (Post) | 0.015 | -0.357, 0.387 | 0.938 |
|  | Post × Target | 0.195 | -0.228, 0.838 | 0.069 |
|  | Post × MCI-HR | 0.207 | -0.289, 0.633 | 0.641 |
|  | Target × MCI-HR | 0.042 | -0.158, 0.242 | 0.681 |
|  | Post × Target × MCI-HR | 0.133 | -0.561, 0.748 | 0.365 |
| **Vergence Time to Peak** | Stimulus (Target) | 0.109 | -0.057, 0.275 | 0.198 |
|  | Time (Post) | 0.036 | -0.136, 0.208 | 0.689 |
|  | Post × Target | -0.120 | -0.451, 0.108 | 0.305 |
|  | Post × MCI-HR | -0.082 | -0.393, 0.118 | 0.321 |
|  | Target × MCI-HR | 0.011 | -0.216, 0.238 | 0.922 |
|  | Post × Target × MCI-HR | 0.248 | -0.121, 0.680 | 0.128 |
| **Pupil Slope Initial** | Stimulus (Target) | 0.138 | -0.030, 0.306 | 0.108 |
|  | Time (Post) | 0.066 | -0.069, 0.201 | 0.346 |
|  | Post × Target | -0.097 | -0.282, 0.167 | 0.418 |
|  | Post × MCI-HR | -0.040 | -0.239, 0.153 | 0.586 |
|  | Target × MCI-HR | 0.057 | -0.172, 0.286 | 0.628 |
|  | Post × Target × MCI-HR | 0.069 | -0.272, 0.380 | 0.681 |
| **Pupil Slope Global** | Stimulus (Target) | 0.821 | 0.664, 0.978 | **<0.001** |
|  | Time (Post) | 0.005 | -0.126, 0.136 | 0.942 |
|  | Post × Target | -0.152 | -0.430, 0.341 | 0.195 |
|  | Post × MCI-HR | -0.011 | -0.164, 0.149 | 0.860 |
|  | Target × MCI-HR | 0.045 | -0.171, 0.261 | 0.683 |
|  | Post × Target × MCI-HR | 0.316 | 0.111, 1.013 | **0.045** |
| **Pupil Slope Late** | Stimulus (Target) | 0.006 | -0.160, 0.172 | 0.940 |
|  | Time (Post) | 0.031 | -0.130, 0.192 | 0.705 |
|  | Post × Target | -0.150 | -0.410, 0.147 | 0.212 |
|  | Post × MCI-HR | -0.038 | -0.249, 0.192 | 0.570 |
|  | Target × MCI-HR | 0.084 | -0.143, 0.311 | 0.466 |
|  | Post × Target × MCI-HR | 0.417 | 0.054, 1.017 | **0.013** |
| **Pupil AUC** | Stimulus (Target) | 0.734 | 0.575, 0.893 | **<0.001** |
|  | Time (Post) | 0.020 | -0.121, 0.161 | 0.781 |
|  | Post × Target | -0.139 | -0.458, 0.157 | 0.226 |
|  | Post × MCI-HR | -0.040 | -0.193, 0.145 | 0.546 |
|  | Target × MCI-HR | 0.040 | -0.178, 0.258 | 0.720 |
|  | Post × Target × MCI-HR | 0.219 | -0.282, 0.717 | 0.168 |
| **Pupil Peak Power** | Stimulus (Target) | 0.091 | -0.058, 0.240 | 0.229 |
|  | Time (Post) | 0.116 | -0.178, 0.410 | 0.447 |
|  | Post × Target | -0.165 | -0.581, 0.191 | 0.110 |
|  | Post × MCI-HR | 0.062 | -0.308, 0.432 | 0.704 |
|  | Target × MCI-HR | 0.048 | -0.154, 0.250 | 0.641 |
|  | Post × Target × MCI-HR | 0.267 | -0.230, 0.808 | 0.073 |
| **Pupil Time to Peak** | Stimulus (Target) | 0.105 | -0.061, 0.271 | 0.213 |
|  | Time (Post) | -0.032 | -0.259, 0.195 | 0.782 |
|  | Post × Target | 0.106 | -0.181, 0.390 | 0.369 |
|  | Post × MCI-HR | -0.064 | -0.512, 0.152 | 0.519 |
|  | Target × MCI-HR | -0.023 | -0.250, 0.204 | 0.843 |
|  | Post × Target × MCI-HR | -0.195 | -0.581, 0.136 | 0.227 |

Reference category: MCI-LR, pre-intervention, Distractor trials. Coefficients are standardized. For two-way and three-way interaction terms, p-values are from Freedman-Lane permutation tests (5,000 permutations) and 95% CIs are from BCa cluster bootstrap (5,000 iterations), resampling participants as the exchangeable unit. For main effect terms, p-values and 95% CIs are Wald-based. Bold p-values indicate statistical significance (p < .05)

Figure S1. Schematic representation of the eye-tracking game and experimental setup.

In the actual task, a single stimulus was presented per trial, consisting of either a target (bullseye) or a distractor (owl). The on-screen cursor reflected the real-time position of the participant's gaze or mouse input.

Figure S2. Oddball Task sequence.

Strings of symbols are presented for two seconds, followed by a grey mask. In 80% of the trials the symbols were printed in blue, and in the remaining 20% they were printed in red.
